# Cinobufacini ameliorates experimental colitis via modulating the composition of gut microbiota

**DOI:** 10.1371/journal.pone.0223231

**Published:** 2019-09-30

**Authors:** Yongfeng Bai, Siwei Wang, Wenkai Xu, Yuanyuan Weng, Shengmei Zhu, Hao Sheng, Jin Zhu, Feng Zhang

**Affiliations:** 1 Department of Clinical Laboratory, Quzhou People's Hospital, Quzhou, Zhejiang, China; 2 Department of Core Facility, Quzhou People's Hospital, Quzhou, Zhejiang, China; 3 Department of Medical Information and Technology, Quzhou People's Hospital, Quzhou, Zhejiang, China; 4 Medical School, Zhejiang University, Hangzhou, Zhejiang, China; National Institute for Agronomic Research, FRANCE

## Abstract

**Background:**

Cinobufacini, the sterilized hot water extraction of dried toad skin, has been widely used in the treatment of inflammation and cancers. Recently we found cinobufacini could ameliorate dextran sulfate sodium (DSS)-induced colitis in mice, but the underlying mechanism was not fully understood. In current study, we explored the effect of cinobufacini on gut microbiota in DSS-induced acute colitic mouse model by pyrosequencing of colonic contents.

**Methods:**

C57BL/6 mice were supplied with normal or 3.0% DSS containing drinking water. DSS-treat mice were gavaged daily either with vehicle (water) or cinobufacini (10.0 or 30.0 mg/kg) for 7 days. The composition of the gut microbiota was assessed by analyzing 16S rRNA gene sequences.

**Results:**

Our data indicated that cinobufacini reversed DSS-induced gut dysbiosis and enhanced intestinal barrier integrity. Moreover, changing of some specific microbial groups such as *Proteobacteria* and *Bacteroides* was closely correlated with the re-establishment of intestinal equilibrium and the recovery of intestinal function.

**Conclusion:**

Cinobufacini prevents colitis in mice by modifying the composition and function of gut microbiota. The current study provides additional mechanistic insight in the therapeutic effect of cinobufacini treatment and may pave the way for clinical application of cinobufacini in colitis therapy.

## Introduction

Inflammatory bowel disease (IBD), including Crohn's disease (CD) and ulcerative colitis (UC), is a heterogeneous group of chronic and relapsing inflammatory disorder of the gut. IBD is common in industrialized countries, but the incidence is increasing rapidly in Asia and South America nowadays[1]. A number of factors, such as immune function, genetics, and environmental factors such as smoking, antibiotics use, diet and so on, are associated with developing IBD[1].

The importance of gut microbiota in the pathogenesis of IBD has attracted more attention during the past decade. A signature of IBD is dysbiosis, characterized by reduced gut microbial diversity because of breakdown of the balances between putative species of “protective” versus “harmful” intestinal bacteria[2]. Antibiotics and probiotics show clinical effects when used for the treatment of IBD[3]. Many of the known IBD susceptibility genes are associated with intestinal mucosal barrier function and are involved in host-microbiota interactions[4]. Other observations support a role for the gut microbiota in IBD including the use of faecal microbiota transplantation (FMT) as a therapeutic approach in IBD, and the rapidly increasing incidence of IBD globally associated with a Westernized lifestyle and other associated environmental factors[1,3,5,6].

5-aminosalicylic acid (5-ASA), an anti-inflammatory chemical, is widely prescribed for the treatment of IBD in clinical practice. The emerging evidences reveal that gut bacteria are the therapeutic targets of 5-ASA[7]. In Asian countries, traditional Chinese medicine (TCM) is also widely used in IBD treatment. A large amount of evidence reveals that TCM plays an essential role in gut microbiota during IBD treatment. Berberine reduces diversity of the gut microbiome and interferes with the relative abundance of *Bacteroides*, *Eubacterium*, and *Desulfovibrio* in the intestine of UC model mice[8]. Curcumin supplementation enhances bacterial richness and diversity and modulates the relative abundance of some orders, including *Lactobacillales* and *Coriobacterales*, in the intestine of colitis-associated colorectal cancer model mice[9].

Cinobufacini (Huachansu), an aqueous extract from the skin of the Bufo toad, is a traditional Chinese medicine widely used in clinic with anti-tumor and anti-inflammatory effects[10]. In previous study, our team found that cinobufacini could relief DSS-induced colitis in mice[11], but the underlying mechanism is still elusive. Bufadienolides, the principal bioactive components of cinobufacini, have been reported to have strong antimicrobial activity in vitro[12]. However, it is unclear whether cinobufacini could influence colitis pathogenesis by regulating gut microbiota. In this study, we explored how cinobufacini reshaped gut microbiome in the context of DSS-induced colitis.

## Materials and methods

### Animal experiments

Male C57BL/6 mice were obtained from Shanghai Laboratory Animal Center, where they were maintained under specific pathogen-free conditions with a 12-h light/dark cycle. The animal experimental procedures were approved by the Committee on the Ethics of Animal Experiments of Zhejiang University of Traditional Chinese Medicine, China. Studies involving animals were performed with compliance to all relevant ethical regulations. To avoid any possible interference from gender, only male mice were used in this study. After 1 week of acclimation, 8-week-old mice were randomly divided into four groups of 8 mice. The control group was supplied with normal drinking water for 7 days, and one group (assigned as DSS group) exposed to drinking water containing 3.0% DSS (36 to 50kDa; MP Biomedicals, USA) for 7 days, whereas the other two groups were fed 3.0% DSS plus 10.0 or 30.0 mg/kg (body weight) cinobufacini by gavage administration for 7 days (assigned as cinobufacini-L group and cinobufacini-H group). Cinobufacini capsule (National drug standard: Z20050846) used for animal experiment was purchased from Shanxi Dongtai Pharmaceutical Co., Ltd. (China). Body weight, stool consistency, and stool bleeding were assessed daily.

At the end of the experiment, the animals were sacrificed under general anesthesia. To detect gut permeability, four mice per group were gavaged with FITC-Dextran (0.6 mg/g body weight; Sigma, China) 4 hours before the fluorometric analysis of FITC fluorescence in serum as described previously^13^. Blood was drawn in EDTA-K2 tubes and immediately centrifuged in order to separate plasma from cells. The samples of colonic content were collected and stored at −80°C for 16S rRNA sequencing. Colon tissues were collected and cutted into three pieces. We used the proximal colon for MPO and pro-inflammatory cytokines (frozen immediately), the middle portion for RNA isolation (frozen immediately), and the rectal region for histology (fixed in 10% formalin in a cassette)[13].

### Evaluation of Colitis and detection of MPO, cytokines in colon tissue and FITC-Dextran in blood

The disease activity index (DAI) of mice was evaluated according to previous studies[13]. To evaluate histological damage of colitis severity, the rectal colon stained with hematoxylin and eosin (H&E) for histopathological analysis. Histopathological scores were determined by a blinded observer using a previously published system[14]. The proximal colon tissue was homogenized. The supernatant was used to quantify the MPO activity using a colorimetric assay according to previous studies[15]. The levels of pro-inflammatory cytokines in peripheral blood were also determined using ELISA kits (MEIMIAN, China). Serum FITC-Dextran was assayed by BioTek Synergy H1 microplate reader (excitation of 488 nm and an emission of 520 nm).

### RNA isolation and quantitative real-time PCR

For quantitative real-time PCR analysis, the middle colonic tissue was homogenized and RNA was extracted using the Trizol method. To remove residual DSS contaminants, we purified the colonic RNAs using lithium chloride protocol[16]. The complementary DNA (cDNA) was synthesized using Reverse Transcriptases kits (Thermo, USA). Realtime PCR was performed on LightCycler 480 instrument (Roche) using SYBR Green (Sangon, China). The relative mRNA expression was analysed using the comparative Ct method. The primers used in this paper are listed in S1 Table. The expression of the GAPDH gene was used as an external control.

### Gut microbiota analysis

To analyze gut microbiota, every six colonic content samples in each group (including the control group, the DSS group and the cinobufacini-H group) were randomly selected and pooled to yield total eighteen samples (n = 6 per group). Total genome DNA from samples was extracted using CTAB/SDS method. The V3–V4 region of 16S rRNA was amplified with universal primers. Sequencing and data analysis were subsequently performed on an Ion S5 TM XL platform by Novogene (Beijing, China) using a method described previously[17]. Briefly, ≧97% similarity of the sequences were classified as the same OTUs. The representative sequence of each OTU was screened for the further annotation.

In order to compute alpha diversity, we rarified the OTU table and calculated two metrics: Chao1 estimated the species abundance; Shannon index accounted for both abundance and evenness of the species present using QIIME software (version 1.7.0). We used weighted unifrac for Principal Coordinate Analysis (PCoA) by R software (Version 2.15.3). In addition, Metastats software (version 1.5) was used to analysis significant differences between different groups. Biomarker discovery using Linear Discriminant Analysis Effect Size (LEfSe) and functional prediction using Reconstruction of Unobserved States (PICRUSt) were performed online (http://huttenhower.sph.harvard.edu/galaxy).

### Statistics analysis

All data were analyzed using one way analysis of variance (ANOVA) followed by Tukey’s test with mean ± SD (standard deviation) for the independent experiments. Statistical differences between different groups were examined using SPSS (version 20.0). P < 0.05 was considered to be statistically significant. The graph was created on GraphPad Prism 6 software.

### Data availability

The raw 16S rRNA sequences for the microbiota analyses were deposited into the NCBI Sequence Read Archive database with accession number PRJNA563762. Any other data that support the findings of this study are available from the corresponding authors upon reasonable request.

## Results

### Cinobufacini ameliorated DSS-induced acute colitis in mice

**Fig 1:**
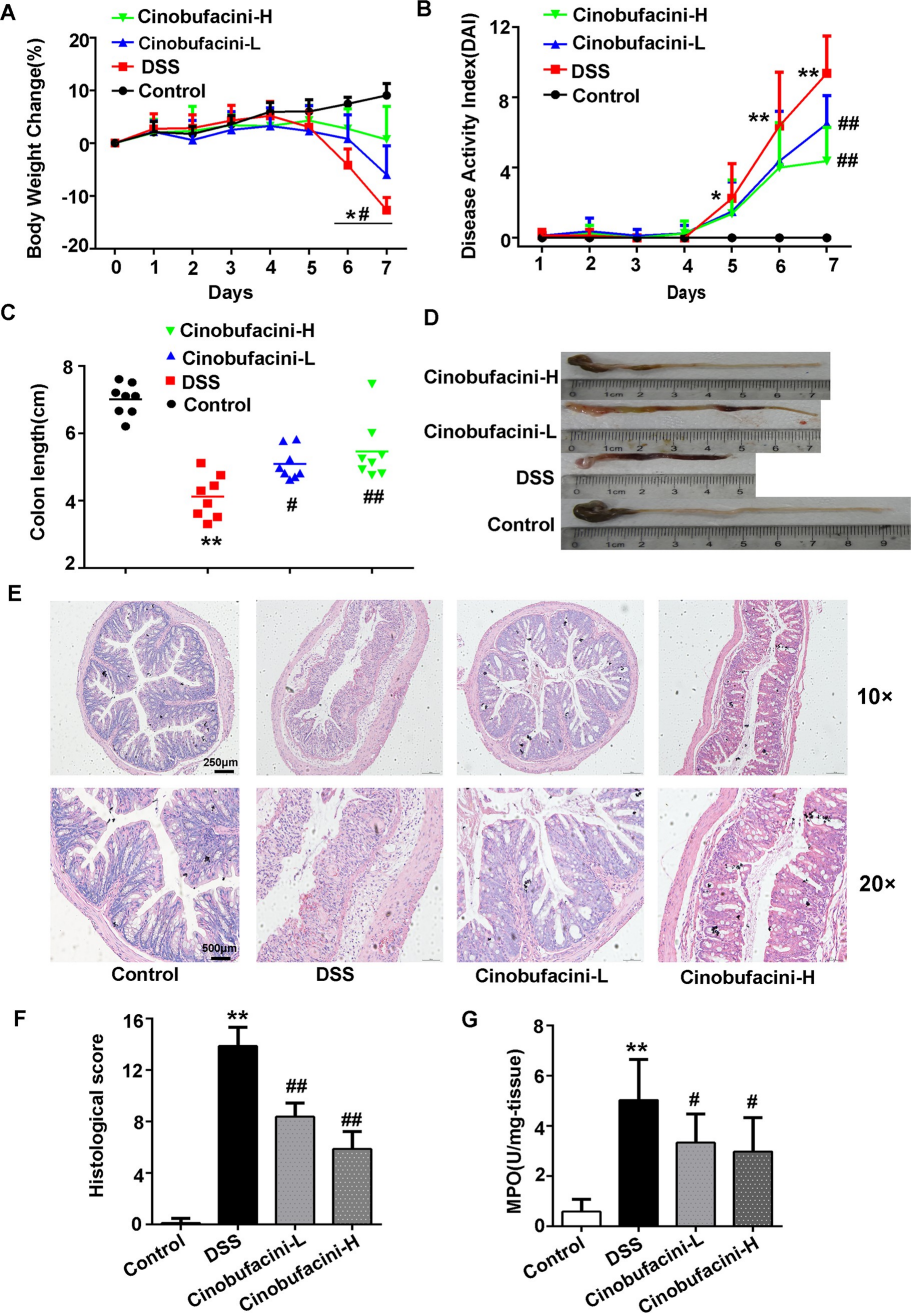
The effect of cinobufacini on DSS-induced colitis in mice. (A) Percent change in body weight. (B) Disease activity index score. (C, D) The change of colon length. (E, F) Representative H&E-stained rectal colonic section and histological score. (G) The myeloperoxidase activity of colonic tissue. Cinobufacini-L means low dose of cinobufacini treatment, which is 10.0 mg/kg body weight; cinobufacini-H indicates high dose of cinobufacini, 30.0 mg/kg body weight. Data shown are the means ± SD. *p < 0.05, **p < 0.01 vs the control group; #p < 0.05, ##p < 0.01 vs the DSS group. n = 8 mice per group.

We researched the therapeutic effect of cinobufacini on DSS-induced acute colitis in C57BL/6 mice at first. Mice receiving 3.0% DSS developed serious colitis characterized by significant weight loss, diarrhea and hematochezia, reflected by the increased disease activity index. Treatment with cinobufacini reduced body weight loss, diarrhea and the blood in feces (Fig 1A and 1B). The average colon length of DSS-treated mice was generally shorter than that of the cinobufacini group (Fig 1C and 1D). From the histopathology, administration of DSS significantly induced the intestinal inflammatory response, manifested as mucosal ulceration, epithelium disruption, and inflammatory cell infiltration, but cinobufacini treatment prominently reduced the severity of histopathologic response (Fig 1E and 1F). MPO activity reflects the infiltration of neutrophil in inflammatory tissue environment[15]. As shown in Fig 1G, the MPO activity was much higher in DSS group compared with the control group, while treatment with cinobufacini obviously lowered DSS induced MPO elevation. Collectively, we conclude that cinobufacini attenuates severity of colitis in DSS-treated mice.

### Cinobufacini decreased the production of inflammatory cytokines and intestinal permeability

**Fig 2:**
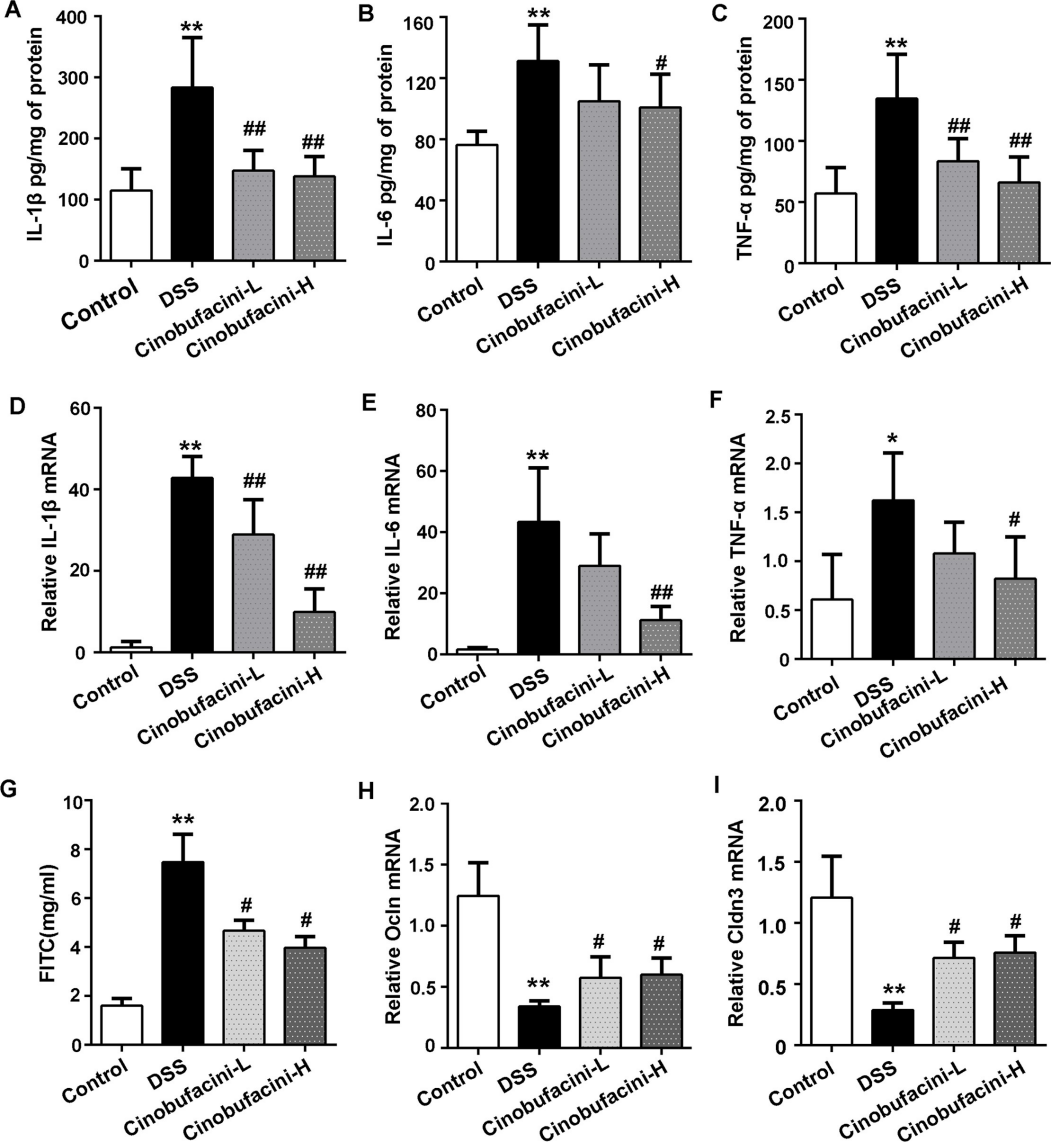
Cinobufacini reduced the production of inflammatory cytokines and intestinal permeability. (A, B, C) Measurement of inflammatory cytokines in mouse blood samples. (D, E, F) mRNA level of inflammatory cytokines in the colon tissues (G) FITC-Dextran permeability testing. (H, I) The expression of key molecular components of tight junctions. Cinobufacini-L, 10.0 mg/kg body weight cinobufacini treatment; cinobufacini-H, 30.0 mg/kg body weight. Data are represented as the mean ± SD of three experimental replicates. *p < 0.05, **p < 0.01 vs the control group; #p < 0.05, ##p < 0.01 vs the DSS group. n = 8 mice per group.

IL-1β plays an important role in the progression of IBD disease and is associated with the severity of intestinal inflammation. In many cases, IL-1β can induce the expression of other pro-inflammatory cytokines such as IL-6 and TNF-α leading to intestinal inflammation[18]. Indeed, in DSS-treated mice, the levels of inflammatory cytokines IL-1β, IL-6 and TNF-α in colon tissue increased markedly compared to the control group. Treatment with cinobufacini significantly suppressed the abundance of IL-1β, IL-6 and TNF-α in the blood of colitic mice (Fig 2A–2C). Meanwhile, the contents of IL-1β, IL-6 and TNF-α were decreased in colonic tissues of cinobufacini-treated mice compared with DSS-treated mice at the mRNA level (Fig 2D–2F).

To further characterize the protective effects of cinobufacini on the barrier function of intestine, we performed intestinal permeability assay using an FITC-labeled dextran method *in vivo*. The leak of FITC-dextran in serum was measured. The result showed that cinobufacini treatment significantly reduced the amount of FITC-dextran in blood compared DSS model mice, suggesting the improvement of intestinal permeability (Fig 2G). The tight junction proteins, including occludin and claudins, play crucial roles in regulating intestinal permeability[19]. We detected the transcription levels of Ocln and Cldn3 in mouse colon tissues by RT-PCR. DSS treatment resulted in a decrease in mRNA expression level of tight junction proteins (Fig 2H and 2I), and it was enhanced in the cinobufacini-treated mice. These data indicate that cinobufacini restores the integrity of intestinal epithelial barrier in DSS-treated mice.

### Cinobufacini regulated intestinal bacterial composition in mice

**Fig 3:**
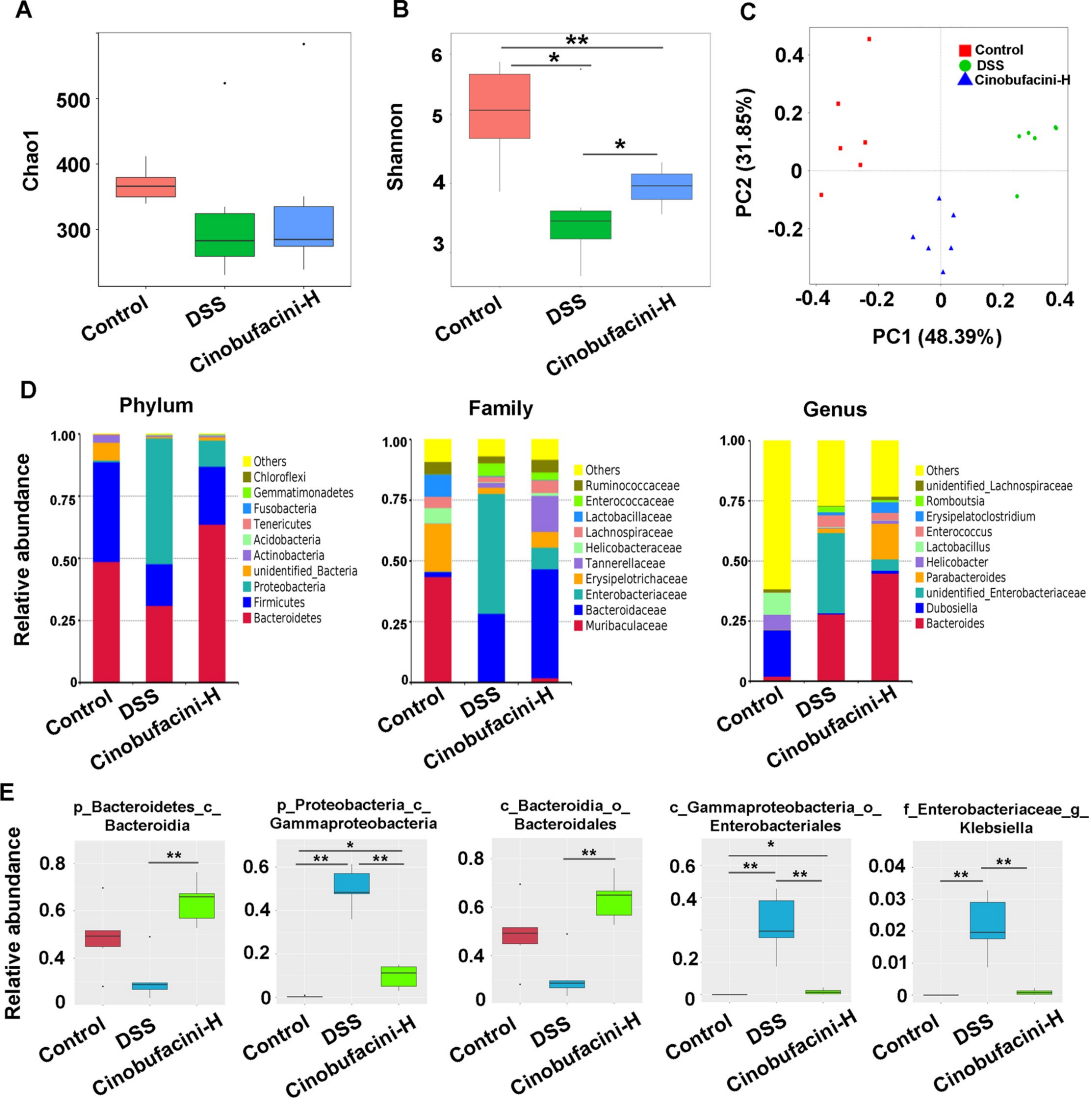
The effect of cinobufacini on the composition of intestinal bacteria in DSS-induced mice. **(**A, B) Alpha diversity indices boxplot, including Chao1 and Shannon. (C) PCoA biplot based on the weighted UniFrac distances. (D) The relative abundance of gut microbiota at phylum, family, and genus levels. (E) MetaStat analysis showing the bacterial abundance significantly reversed by cinobufacini across the different groups. k, kingdom; p, phylum; c, class; o, order; f, family; g, genus. The dose of cinobufacini was 30.0 mg/kg body weight. All values are mean ± SD (n = 6 mice/group). * p <0.05; ** p <0.01.

The impact of cinobufacini on intestinal flora composition was examined by analyzing bacterial 16S rRNA (V3–V4 region). Species richness estimates (Chao1) and diversity indices (Shannon) are presented. Surprisingly, there were no significant differences in the alpha diversity of the communities based on Chao1 index. While, Shannon index indicated that the DSS treatment group was lower than the other two groups (Figs 3A and 2B). We used principal co-ordinates analysis (PCoA) to investigate the community structure of microbiota in three groups. We found that samples tended to cluster together based on different treatment methods. The gut microbiota obtained from the DSS group mostly distinct from those of the other two groups, which indicated obvious modification of the bacterial structure of mouse intestine. The gut microbiota of the cinobufacini group was closer to the control group in PCoA plot (Fig 3C). The change of intestinal bacterial composition among different groups were reflected on the levels of phylum (p), class (c), order (o), family (f), genus (g) and species(s) (Fig 3D; S1 and S2 Figs). In the DSS group, we saw a significant decrease in the abundance of o-*Bacteroidales* (within c-*Bacteroidia*, p*-Bacteroidetes*) and an elevated abundance of g-*Klebsiella*, g-*Proteus* and g-*Enterobacter* (within o-*Enterobacteriales*, c-*Gammaproteobacteria*). In contrast, cinobufacini resulted in the correction of these bacterial groups, which may contribute to the re-establishment of intestinal equilibrium (Fig 3E; S3 Fig).

### Cinobufacini changed biomarkers in each group

**Fig 4:**
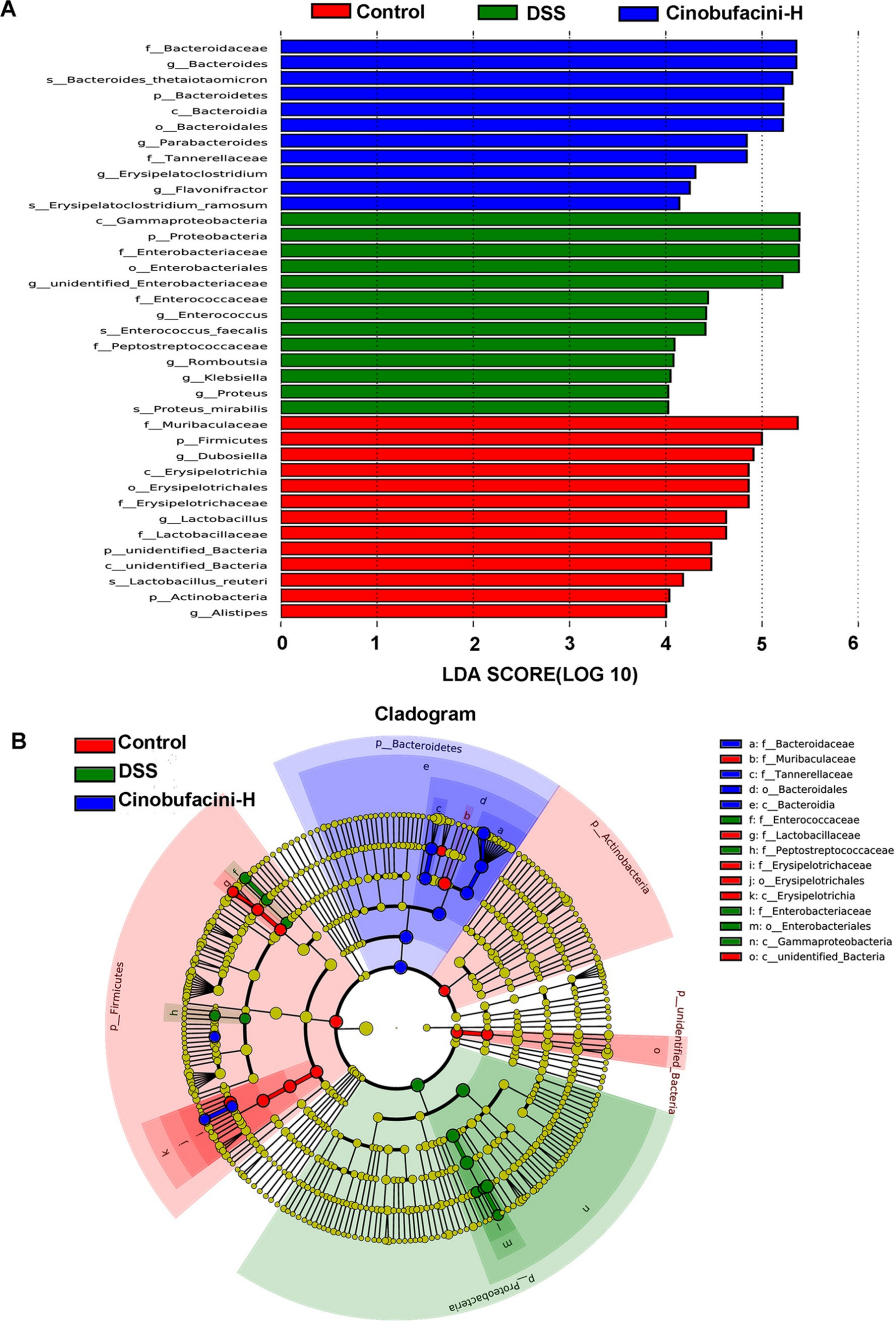
The significantly enriched bacterial taxa in different groups as determined by LEfSe analysis. (A) LEfSe analysis shows differentially abundant bacterial taxa in gut microbiota of different groups (LDA sore >4). (B) LEfSe taxonomic cladogram. The size of each node represents the relative abundance of the species. (p, phylum; c, class; o, order; f, family; g, genus; s, species). The dose of cinobufacini was 30.0 mg/kg body weight. All values are mean ± SD (n = 6 mice/group).

Next, we used LEfSe to detect bacterial organisms differentially abundant among the three groups. The genera *Dubosiella*, *Lactobacillus*, *Alistipes* were biomarkers in the control group. *Enterococcus*, *Romboutsia*, *Klebsiella* and *Proteus* were the dominant phylotypes detected in the DSS group, contributing to the differences between the intestinal microbiota of the control and DSS groups. While the genera *Bacteroides*, *Parabacteroides*, *Erysipelatoclostridium* and *Flavonifractor* were predominant in the cinobufacini group (Fig 4A and 4B). In summary, our results showed that cinobufacini could modulate gut microbiota composition in UC model mice.

### Microbial metabolic functions associated with cinobufacini treatment in DSS-induced colitis

**Fig 5:**
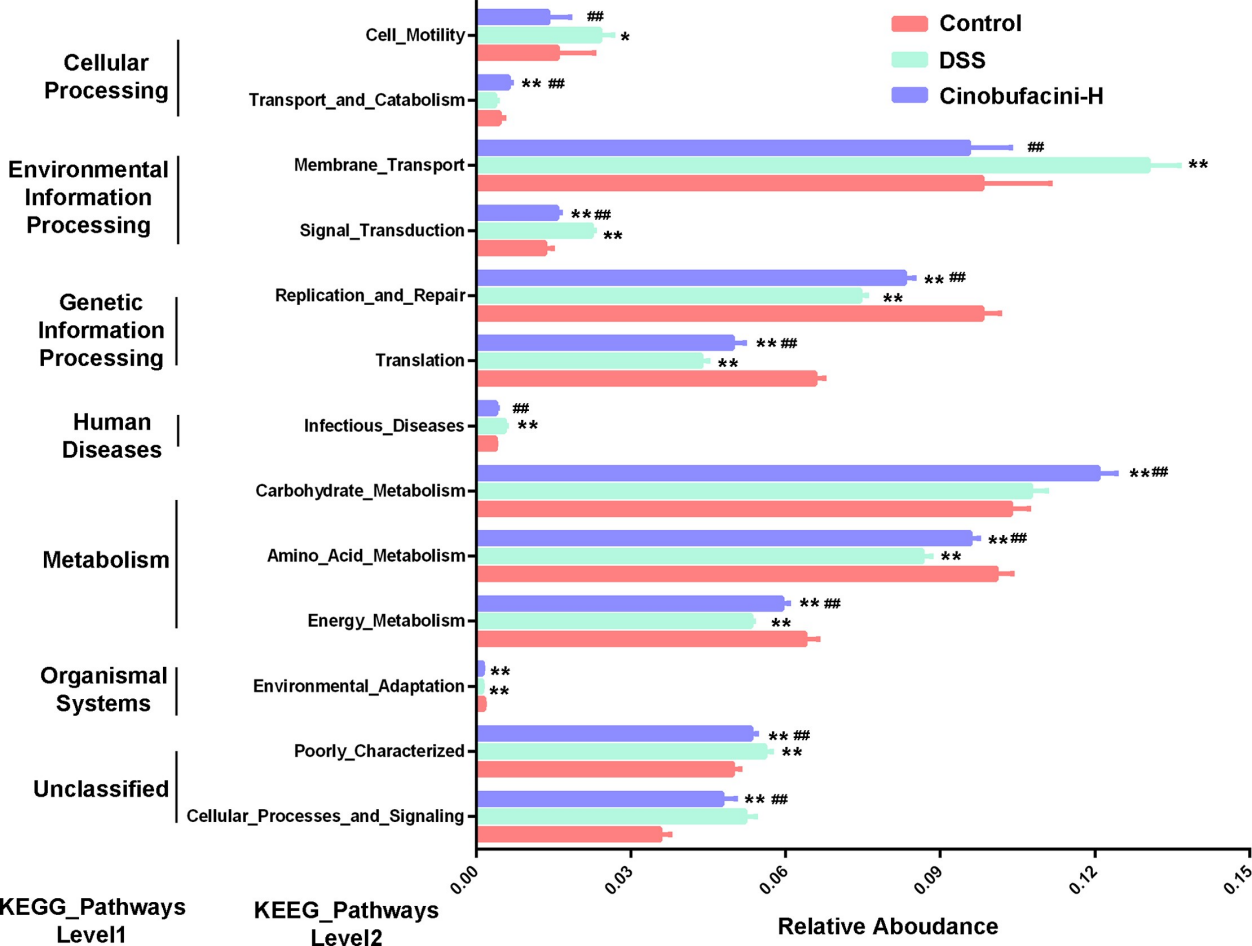
PICRUSt prediction of the functional composition among different mice groups. There are five categories in level 1 KEGG pathway (metabolism, genetic information processing, environmental information processing, cellular processes and human diseases). Relative abundances of most abundant microbial pathways at level 2 in each category among the different groups. The dose of cinobufacini was 30.0 mg/kg body weight. Data shown are the means ± SD (n = 6). *p < 0.05, **p < 0.01 vs the control group; #p < 0.05, ##p < 0.01 vs the DSS group.

To characterize the distinction of functionality of colonic microbiota under DSS-induced and cinobufacini-treated conditions, we used PICRUSt analysis combined with the Kyoto Encyclopedia of Genes and Genomes (KEGG) database of microbial genomic information. The analysis of level 1 KEGG pathways showed a high enrichment of predicted functions related to metabolic pathways, genetic information processing and environmental information processing (S4 Fig). Fig 5 displays the top relative enrichment changes of level 2 KEGG pathways among the different groups. The activity of basic metabolisms, such as amino acid metabolism and energy metabolism, were decreased in the DSS group compared with the control and cinobufacini group. The carbohydrate metabolism was significantly increased in the cinobufacini group compared to the control and DSS group, while there was no difference between the control group and the DSS group (Fig 5). Above all, the analysis of basic metabolisms suggested that the energy output derived from gut microbiota in the DSS group was reduced compared to the control group. The treatment of cinobufacini increased the overall metabolic activity of gut microbiota. Additionally, dysregulated environmental information processing pathways, including membrane transport and signal transduction, were also observed in the DSS group compared with the control group, while treatment of cinobufacini reduced their activities to the control level (Fig 5).

Our results indicated that DSS administration resulted in dysfunction of microbiota in gut. Cinobufacini modulated gut microbiota in DSS-induced colitic mice and recovered the microbial functions close to the level of the control group. These pathways were further analyzed in KEGG level 3 (S5 Fig).

## Discussion

Alterations or dysregulation in the microbiota composition are being correlated to an increased number of diseases, including IBD. In the present study, we examined therapeutic effect of cinobufacini in mice with DSS-induced colitis. Our results indicated that the C57BL/6J mice administrated with DSS developed similar symptoms as the human CD, evidenced by a reduction in body weight loss, DAI score, shortening of colon length, histopathological score as well as infiltration of inflammatory cells. Meanwhile, the results of the cinobufacini group indicated that cinobufacini treatment could alleviate inflammation compared with the DSS group. This finding is consistent with our previous study[11].

Decreased richness or diversity of bacterial species has been reported widely in fecal samples of IBD human patients and DSS-induced colitic rats[20]. In our study, DSS treatment resulted in a significant decline in community diversity (Shannon), but no effect on community richness (Chao1). Meanwhile, the gut microbial communities of the DSS group were clustered together and away from the control group, indicating obvious difference between two groups. However, cinobufacini supplementation recovered the community diversity of the gut and significantly shifted the gut microbiota structure in the PC1 direction. Together these observations indicate that cinobufacini may help the gut microbiota to preserve their community composition and community diversity.

The deleterious roles of various members of the phylum *Proteobacteria* toward the intestinal damage and development of IBD have been well documented. *Proteobacteria* adhesion to and invasion of intestinal epithelial cells might destroy host defenses, stimulate inflammatory response, alter the intestinal microbiota in favor of dysbiosis and ultimately cause IBD[2,21]. For example *Campylobacter*, *Escherichia coli* and *Helicobacter*, have all been associated with the development of IBD[6,21]. In accordance with these previous studies, we demonstrated that the abundance of *Proteobacteria*, mainly including *Klebsiella*, *Proteus* and *Entrobacter*, was suppressed in the gut of model mice received cinobufacini. We suppose the antimicrobial activity against *Proteobacteria* contributes to the therapeutic effect of cinobufacini to colitis.

Intestinal epithelial barrier dysfunction and increased permeability have been described as crucial features in patients with IBD[19,22]. Tight junctions (TJ) in intestinal epithelial cells are involved in regulating the permeability of the intestinal barrier. Studies have shown that *Proteobacteria* can influence intestinal barrier function by regulating the expression and distribution of TJ proteins through various intracellular pathways[21]. *Vibrio cholerae* secretes hemagglutinin/protease to cleave TJ protein occludin. Enteropathogenic *E*. *coli* (EPEC) directly attaches to the surface of intestinal epithelial cells and injects effector proteins into host cells through a type III secretion system (TTSS) to disrupt cellular structures including TJ proteins[22]. In our study, the increased proportion of *Proteobacteria* in the DSS treatment group might be closely related to the reduction of TJ proteins. The administration of cinobufacini reduced the presence of *Proteobacteria* in DSS-induced colitic mouse model and increased occludin and claudins expression. This result suggests that cinobufacini may affect the barrier function of tight junctions by changing intestinal bacteria.

The genus *Bacteroides* is recognized as a dominant and biologically important group of commensal bacteria in the microbiota of the human gastrointestinal tract. *Bacteroides* has been associated with host’s immunoregulatory, metabolic and homeostatic functions[23]. For example, *Bacteroides thetaiotaomicron*, an abundant member of *Bacteroides* genus, has protective effects in both DSS-induced and IL10KO rodent models[24]. A meta-analysis suggests that lower levels of *Bacteroides* are associated with IBD, especially in active phase[25]. This observation was validated by our DSS-induced mouse model. Moreover, cinobufacini treatment significantly increased the abundance of *Bacteroides*. Therefore, the attenuation of inflammatory responses and the reduction of symptoms in CD mouse model treated by cinobufacini could be due to the increase of *Bacteroides*, at least in part.

Functional alterations in gut microbiota resulting from dysbiosis have been consistently shown to be associated with IBD. Under inflammatory state, the bacteria in gut are prone to utilize nutrients from the ambient environment instead of producing nutrients by their own to maintain homeastasis[26]. Indeed, we observed the metabolic function predicted by PICRUSt algorithm decreased in the microbiota from DSS-induced colitic mice compared to control group. It is consistent with the previous study showed the biosynthesis of amino acids, butanoate and histidine metabolism was decreased in the DSS-induced UC mode[8]. In contrast, the bacteria need to sense the changes of the surrounding environment for survival purpose under dramatic environmental change such as severe inflammatory responses[27]. We found the functionality for environmental information processing increased in the DSS group, probably for fulfilling this specific demand under inflammatory state. The change of gut microbiota by cinobufacini treatment also recovered the functions of gut bacteria. Carbohydrate fermentation by *Bacteroides* results in the production of a pool of volatile fatty acids that are reabsorbed through the large intestine and utilized by the host as an energy source, providing a significant proportion of the host's energy requirement[23]. The increase in *Bacteroides* in cinobufacini treatment group may play an important role in the recovery of intestinal function. Taken together, the predicted functionality recovery further demonstrated the underlying mechanism of cinobufacini treatment in colitis.

The shortcoming of our investigation is lack of detailed insight on the interaction between cinobufacini and gut microbiota. There is few literature reporting cinobufacini has anti-bacterial activity in other diseases. It is infeasible to create a culture mode in vitro to examine the anti-bacterial activity of cinobufacini, since most of the bacteria changed by cinobufacini administration are unculturable. Furthermore, it is also possible that cinobufacini exerts its impact on gut microbiota through signaling pathways in enterocyte and immune cells resident in intestine. For example, NLRP3 and NLRP6 affect the nature of the flora that inhabits the intestine[28]. Whether cinobufacini modulates gut microbiota directly or via intracellular signaling pathway like NLRP3 needs further study.

Growing evidence suggests that nuclear factor (NF)-κB signaling plays a significant role in intestinal inflammatory disorders[29]. Certain bacteria could activate or inhibit NF-κB signaling pathway. Kostic et al. demonstrated that Fusobacterium nucleatum can instigate NF-κB signaling pathway to induce intestinal tumorigenesis[30]. It was also reported that Lactobacillus rhamnosus, a species of protective bacteria, suppressed the expression of inflammatory proteins NF-κB p65 and induced the expression of p53 and BAX to prevent colon cancer development[31]. In our previous studies[11], it was also demonstrated that cinobufacin can inhibit the activation of the NF-κB pathway, but whether it is directly affected by the alteration of specific microflora requires further exploration.

In summary, our study demonstrates that cinobufacini could modulate the composition of the intestinal flora and restore the relative abundances of vital bacteria including *Klebsiella*, *Proteus*, *Entrobacter* and *Bacteroides*, preventing the imbalance of gut microbiota in DSS-induced colitis. The current study provides additional mechanistic insight in the therapeutic effect of cinobufacini treatment and may pave the way for clinical application of cinobufacini in colitis therapy.

## Supporting information

S1 Fig The effect of cinobufacini on the composition of intestinal bacteria in DSS-induced mice at order and class levels. The dose of cinobufacini was 30.0 mg/kg body weight.
(TIF)

S2 Fig Heatmap illustrations of gut bacterial taxa changes at phylum, family and genus levels. The color intensities indicate the relative abundance of bacterial taxa in each group. The dose of cinobufacini was 30.0 mg/kg body weight.
(TIF)

S3 Fig MetaStat analysis showing the bacterial abundance significantly reversed by cinobufacini across the different groups. k, kingdom; p, phylum; c, class; o, order; f, family; g, genus. The dose of cinobufacini was 30.0 mg/kg body weight. All values are mean ± SD (n = 6 mice/group). * p <0.05; ** p <0.01.
(TIF)

S4 Fig KEGG pathway functional classification and annotation. (TIF)

S5 Fig Clustered heatmap of KEGG pathway (Level 3) enrichment analysis. The color intensities indicate enrichment score of each KEGG pathway. The dose of cinobufacini was 30.0 mg/kg body weight.
(TIF)

S1 Table The primers used in this article. (DOCX)
