# Supplementary figures and images for: Cinobufacini ameliorates experimental colitis via modulating the composition of gut microbiota
Source: PLoS One. 2019 Sep 30;14(9):e0223231. doi: 10.1371/journal.pone.0223231 (PMC6768468; doi:10.1371/journal.pone.0223231)

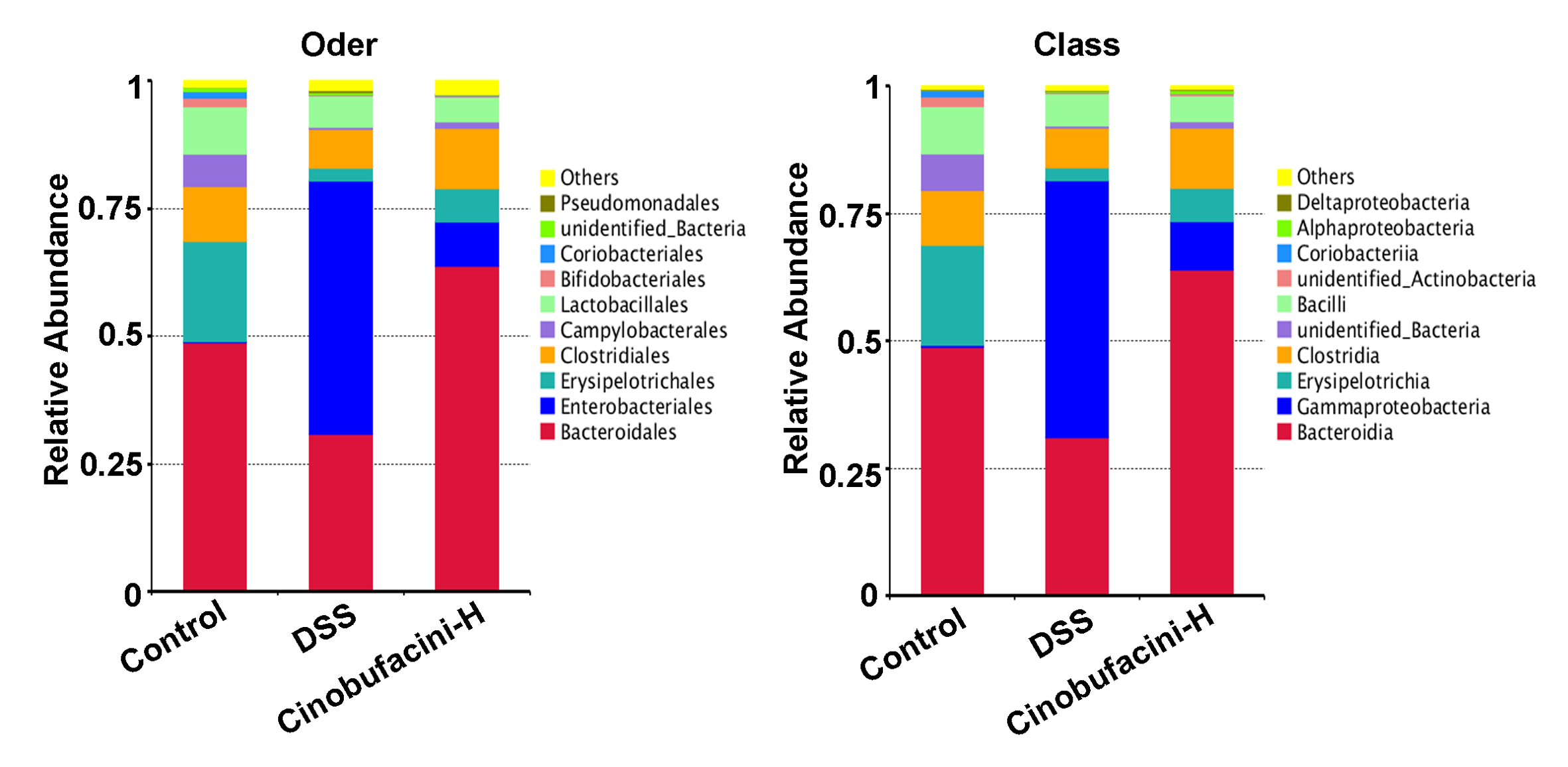

Supplement: S1 Fig — The dose of cinobufacini was 30.0 mg/kg body weight. (TIF) [file pone.0223231.s001.tif]

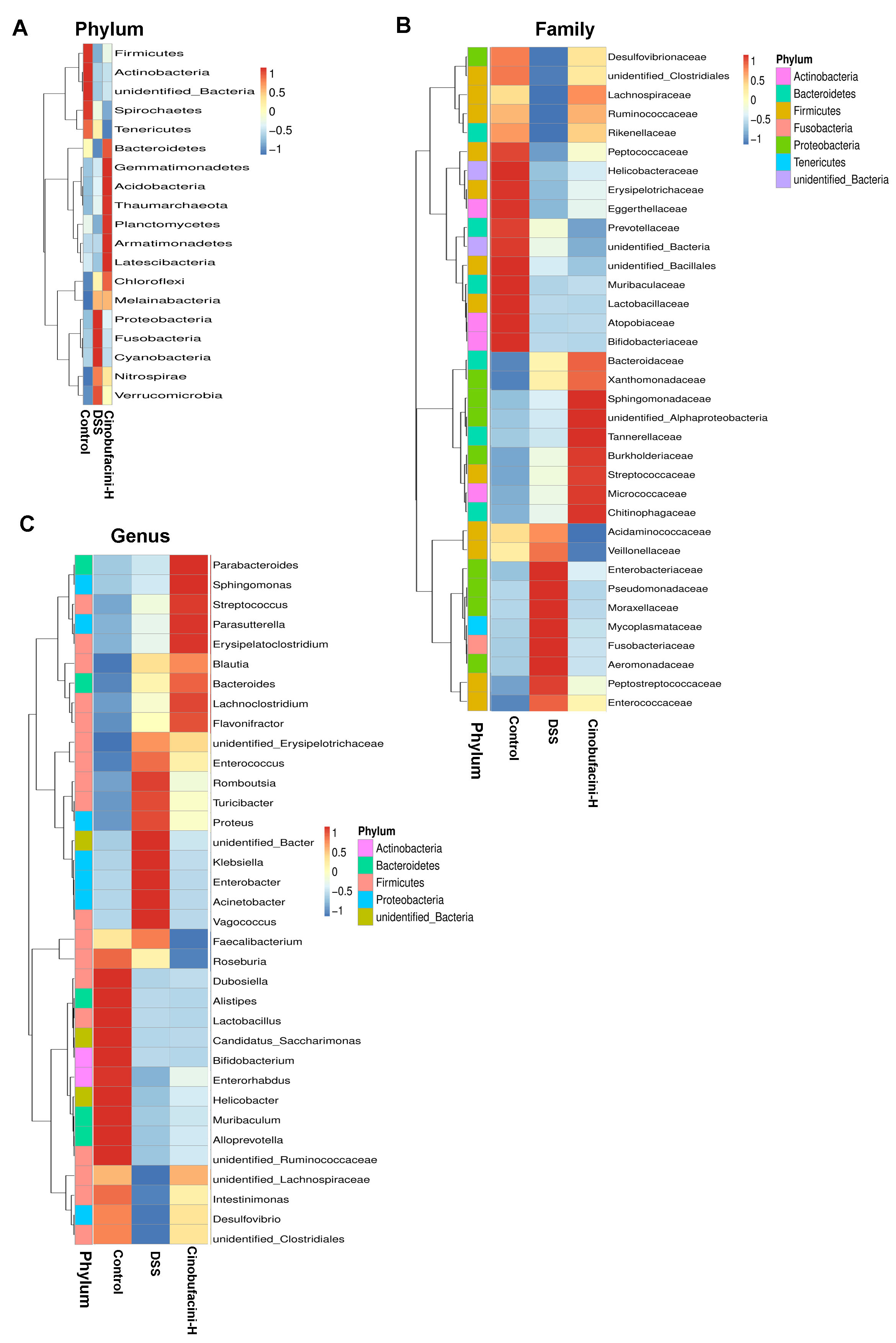

Supplement: S2 Fig — The color intensities indicate the relative abundance of bacterial taxa in each group. The dose of cinobufacini was 30.0 mg/kg body weight. (TIF) [file pone.0223231.s002.tif]

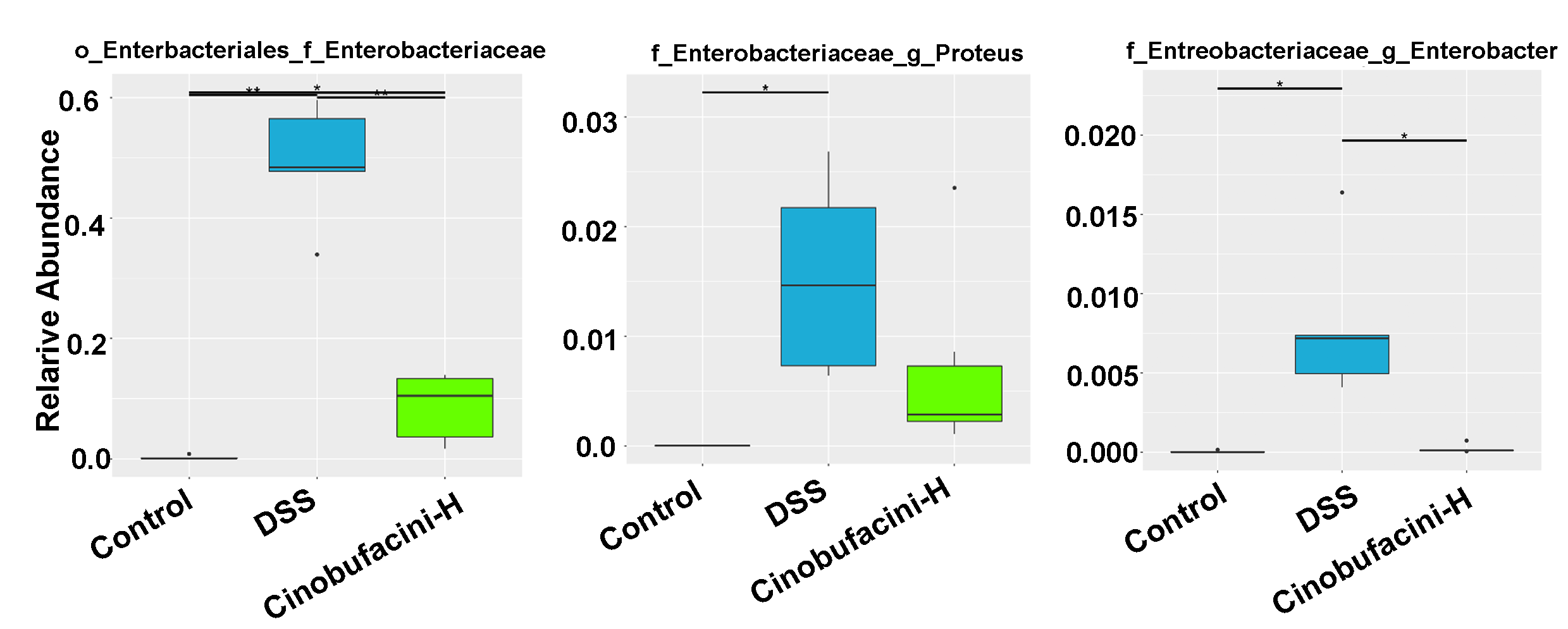

Supplement: S3 Fig — k, kingdom; p, phylum; c, class; o, order; f, family; g, genus. The dose of cinobufacini was 30.0 mg/kg body weight. All values are mean ± SD (n = 6 mice/group). * p <0.05; ** p <0.01. (TIF) [file pone.0223231.s003.tif]

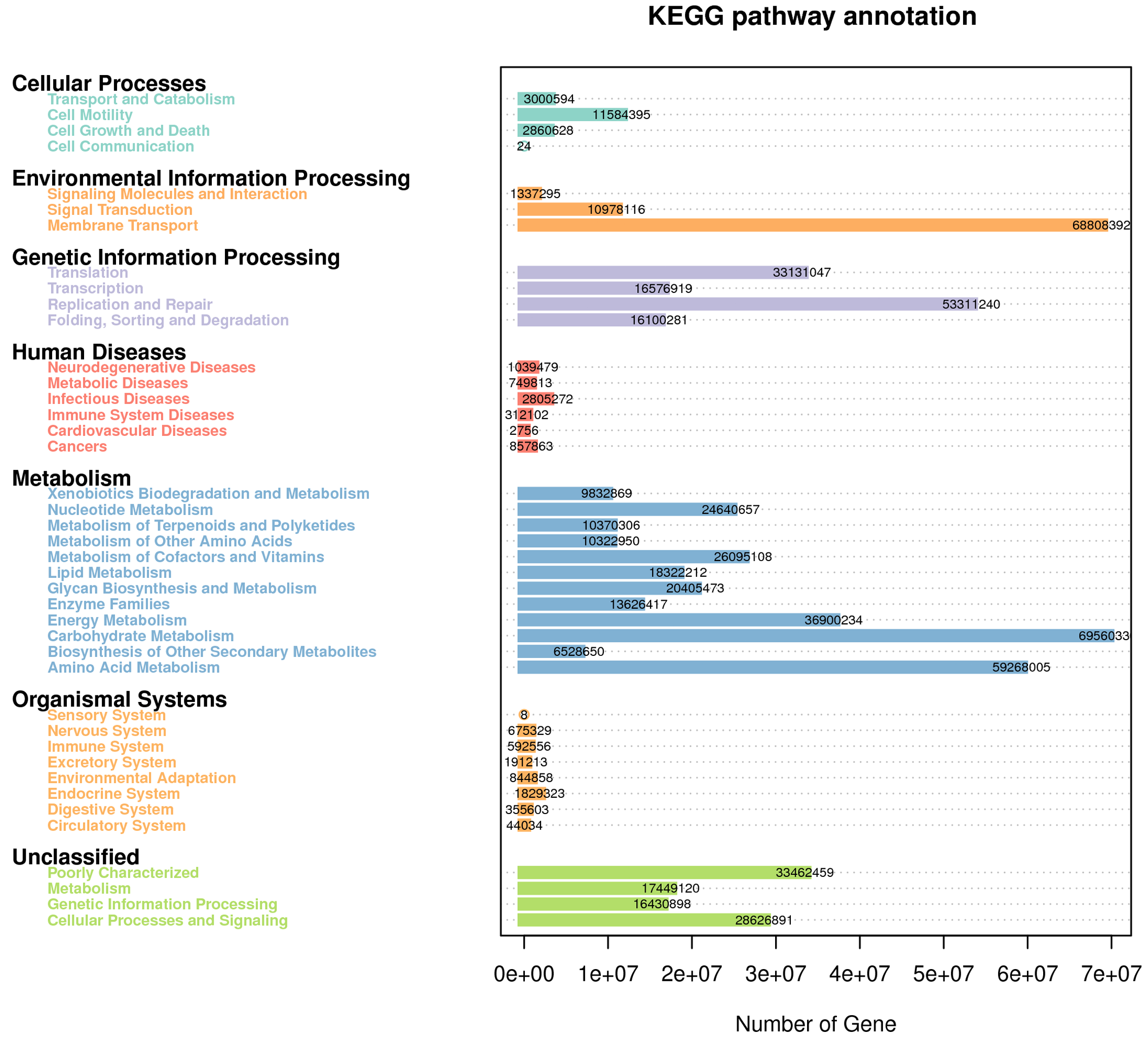

Supplement: S4 Fig — (TIF) [file pone.0223231.s004.tif]

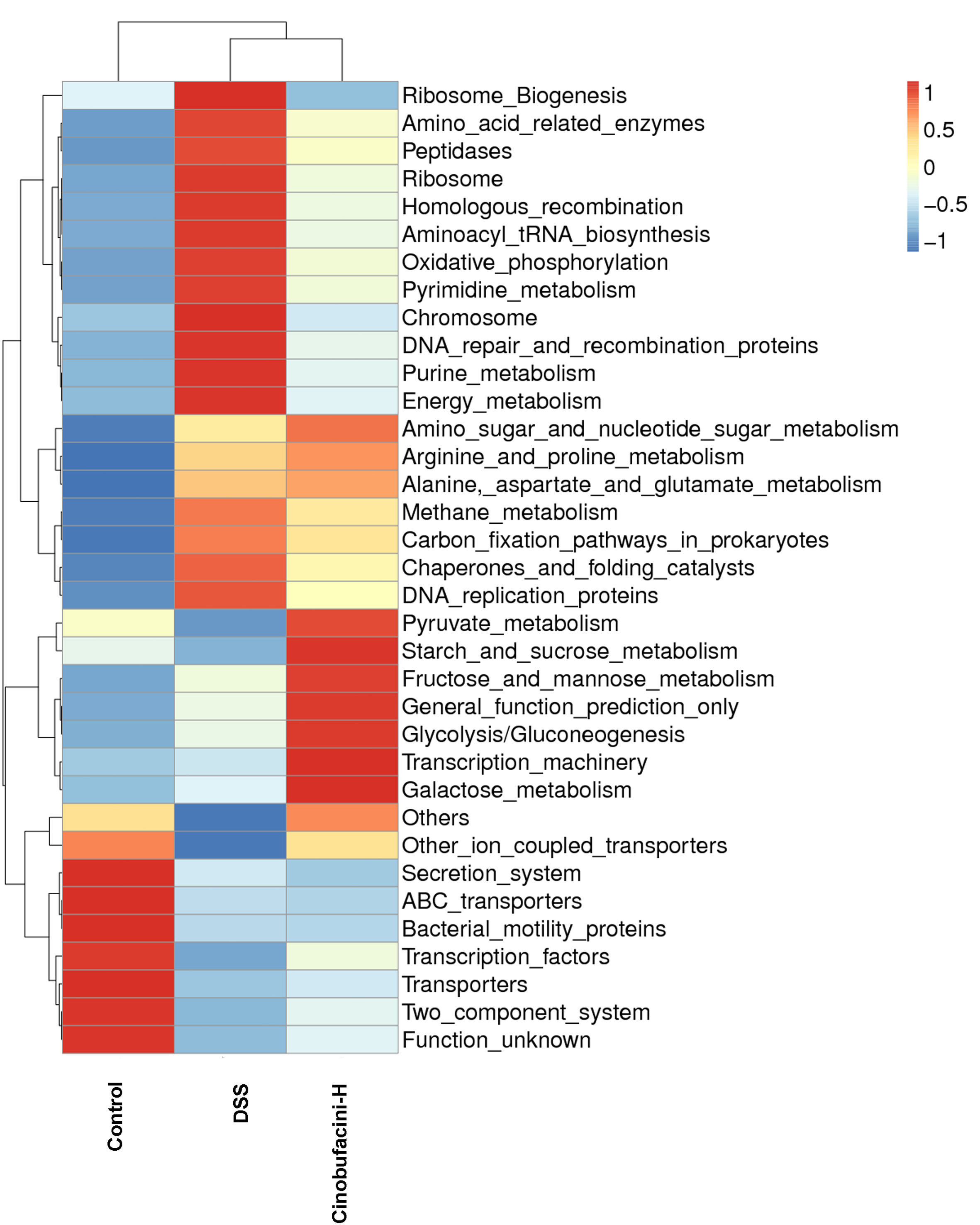

Supplement: S5 Fig — The color intensities indicate enrichment score of each KEGG pathway. The dose of cinobufacini was 30.0 mg/kg body weight. (TIF) [file pone.0223231.s005.tif]
