# Supplementary material for: Cinobufacini ameliorates experimental colitis via modulating the composition of gut microbiota
Source: PLoS One. 2019 Sep 30;14(9):e0223231. doi: 10.1371/journal.pone.0223231 (PMC6768468; doi:10.1371/journal.pone.0223231)
Supplement: S1 Table — (DOCX) [file pone.0223231.s006.docx]

S1 Table.The primers used in this study for real time PCR

| Description | Sense primer (5’-3’) | Antisense primer (5’-3’) |
| --- | --- | --- |
| IL-6 | CTGCAAGAGACTTCCATCCAG | AGTGGTATAGACAGGTCTGTTGG |
| TNF-α | CTGAACTTCGGGGTGATCGG | GGCTTGTCACTCGAATTTTGAGA |
| IL-1β | GAAATGCCACCTTTTGACAGTG | TGGATGCTCTCATCAGGACAG |
| Ocln | ATGTCCGGCCGATGCTCTC | TTTGGCTGCTCTTGGGTCTGTAT |
| Cldn3 | CAGGGGCAGTCTCTGTGCGAG | GCCGCTGGACCTGGGAATCAAC |
| GAPDH | TGAGGCCGGTGCTGAGTATGT | CAGTCTTCTGGGTGGCAGTGAT |
